# A Magnetic-Responsive Biomimetic Nanosystem Coated with Glioma Stem Cell Membranes Effectively Targets and Eliminates Malignant Gliomas

**DOI:** 10.34133/bmr.0123

**Published:** 2024-12-27

**Authors:** Song Deng, Dekang Nie, Yue Huang, Yu Yang, Qianqian Liu, Zesheng Sun, Qiaoji Jiang, Yuejuan Ling, Ya Wen, Jiahua Qu, Jialiang Lin, Yi Wang, Rongqin Huang, Jinlong Shi

**Affiliations:** ^1^ Department of Neurosurgery, Affiliated Hospital of Nantong University, Medical School of Nantong University, Nantong, Jiangsu 226001, P.R. China.; ^2^ Department of Neurosurgery, The Yancheng Clinical College of Xuzhou Medical University, The First People’s Hospital of Yancheng, Yancheng, Jiangsu 224001, P.R. China.; ^3^ Department of Neurology, Affiliated Hospital of Nantong University, Medical School of Nantong University, Nantong, Jiangsu 226001, P.R. China.; ^4^ Institute of Pain Medicine and Special Environmental Medicine, Nantong University, Jiangsu 226019, P.R. China.; ^5^ Research Center of Clinical Medicine, Affiliated Hospital of Nantong University, Nantong, Jiangsu 226001, P.R. China.; ^6^ Center for Advanced Low-Dimension Materials, State Key Laboratory for Modification of Chemical Fibers and Polymer Materials, College of Chemistry, Chemical Engineering and Biotechnology, Donghua University, Shanghai 201600, P.R. China.; ^7^ School of Pharmacy, Key Laboratory of Smart Drug Delivery (Ministry of Education), Fudan University, Shanghai 201203, P.R. China.

## Abstract

Glioblastoma multiforme (GBM) is among the most challenging malignant brain tumors, making the development of new treatment strategies highly necessary. Glioma stem cells (GSCs) markedly contribute to drug resistance, radiation resistance, and tumor recurrence in GBM. The therapeutic potential of nanomaterials targeting GSCs in GBM urgently needs to be explored. A magnetic-responsive biomimetic nanosystem (FDPM), coated with glioma stem cell membranes (CMs), was designed for the targeted eradication of GSCs as well as their associated tumor cells. Identified nanobodies were extensively characterized with various assays. The application tests on nanomaterials were conducted in vitro and in vivo. The tumor-suppressive effects of the nanosystem were evaluated in vitro and in vivo. FDPM can be artificially directed under magnetic guidance while inheriting various biological functions from CM. Upon intravenous injection, FDPM was drawn to the tumor site by magnetic attraction, where it could cross the blood–brain barrier aided by CM. Its homologous targeting ability originates from active proteins on CM, enabling it to specifically target GSCs and related tumor cells. The encapsulated doxorubicin (DOX) within the nanoparticle then destroyed these tumor cells. FDPM demonstrated excellent biocompatibility and tumor-targeting efficiency, effectively targeting malignant gliomas initiated by GSCs. FDPM significantly reduced tumor cells, inhibited tumor growth, and notably extended the survival of glioma-bearing nude mice. The findings position FDPM as a promising nanoplatform to target GSCs and related tumor cells for improving the therapeutic effect of glioma.

## Introduction

Glioblastoma multiforme (GBM), which accounts for approximately 46.1% of all primary malignancies in the brain, has a poor prognosis and a high recurrence rate [1]. With the development of neurosurgery, neuroimaging technology, and intraoperative imaging technology, GBM is now being subjected to comprehensive treatments, including maximum tumor resection, radiation therapy, and adjuvant chemotherapy [2,3]. However, these treatments are still far from the fundamental prevention of the GBM recurrence since GBM is highly invasive and often leaves small tumor satellite lesions after surgery [4,5].

Glioma stem cells (GSCs) play an incredibly critical role in the recurrence of GBM [6–8]. Just like adult stem cells or normal somatic cells that distributed in different tissues, GSCs are considered to be a group of cells that undergo less differentiation in gliomas and are usually in a stationary state [9–11]. However, these GSCs can develop resistance to chemotherapy drugs and ionizing radiation. Also, they have the ability to generate heterogeneous tumor populations [12,13]. Usually, tumor cells without drug and radiation resistance can be maximally cleared via different treatments. Nevertheless, GSCs persist after treatments and proliferate when conditions allow, leading to recurrent solid tumors and a malignant prognosis [14–16]. Therefore, in the routine glioma treatment, if these drug-resistant and radiation-resistant “seed cells” can be targeted and eliminated, it will undoubtedly greatly improve the treatment efficiency of glioma [17–19]. Unfortunately, the targeting strategies against GSCs are scarce during current GBM treatments.

Cancer cells naturally have the capability to adhere to their homologous cells, a process known as homologous adhesion [20,21]. Although the mechanism of homologous adhesion is not yet fully understood, specific proteins or adhesion-related molecules presented on the cell membrane surface play a crucial role. Recently, it has been reported that cancer cell membranes can achieve homologous targeting to corresponding tumor cells [22–24]. Accordingly, targeting drug delivery could be realized by using tumor cell membranes. Since tumor stem cells are the source of tumor cells, their surfaces may also cover the unique adhesion proteins or adhesion molecules possessed by ordinary tumor cells [25–28]. If glioma stem cell membranes (CM) are used to disguise drugs, they may have the potential to target both GSCs and ordinary tumor cells simultaneously [29,30].

**Fig. 1.**
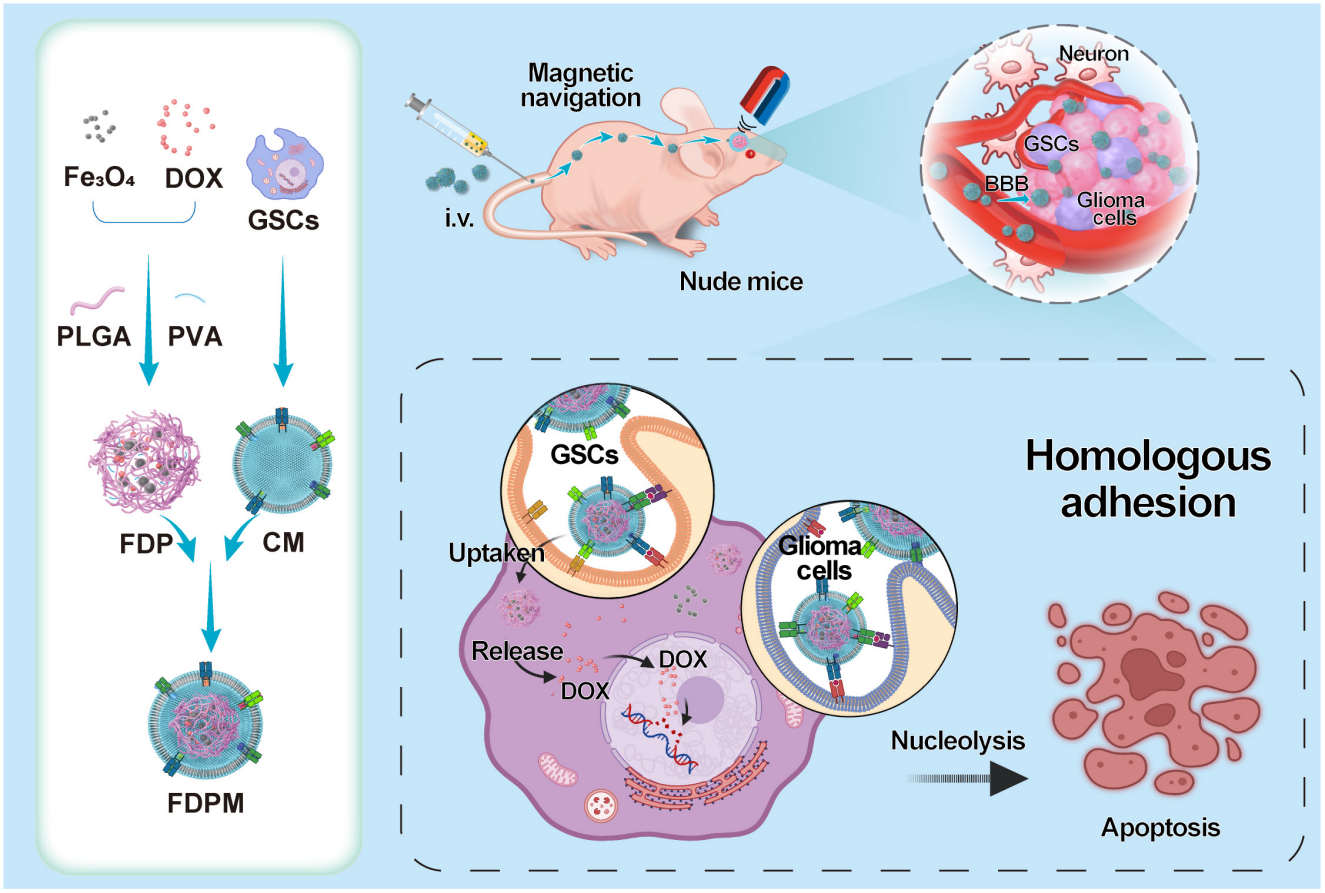
The schematic diagram shows the key steps involved in the preparation of FDPM and its main mechanistic pathways related to malignant glioma treatment.

In this study, a biomimetic nanosystem disguised with CM was constructed for targeted drug delivery against gliomas. As shown in Fig. 1, magnetic ferrosoferric oxide (Fe_3_O_4_) and doxorubicin (DOX) were encapsulated into poly (lactic-co-glycolic acid)-polyvinyl alcohol (PLGA-PVA) nanoparticles to obtain Fe_3_O_4_/DOX@PLGA-PVA (FDP). Then, FDP was coated with CM to obtain the biomimetic nanoparticle of Fe_3_O_4_/DOX@PLGA-PVA@CM (FDPM). Owing to the magnetic targeting, FDPM can decrease the carrying away with blood flow, thus possessing an elevated accumulation in the brain via magnetic field guiding. Meanwhile, tumor cell membranes inherit the immune escape, homologous targeting, and blood–brain barrier (BBB) penetration abilities shown by tumor cells; because of these properties of cell membranes, this FDPM nanoparticle can effectively carry DOX across the BBB and reach the tumor area to exert therapeutic effects [31–34]. The design of this dual-targeted biomimetic nanosystem targeting “seed cells” combined the advantages of 2 targeting effects, allowed nanomaterials to inherit the abilities of tumor cell membranes while also achieving artificial intervention in material enrichment sites, and enabled the nanosystem to play its maximum role in tumor treatment. On the animal model of glioma caused by small amounts of GSCs, the FDPM treatment can effectively suppress tumor growth and significantly extend the average survival time of glioma-bearing mice.

## Materials and Methods

### Preparation of FP, FDP, and FDPM

First, 2 mmol of acetylacetone iron, 10 mmol of 1,2-cetanediol, 3 mmol of hexadecylamine, and 3 mmol of lauric acid were added to 20 ml of dibenzyl ether, stirred and mixed evenly, and then reacted at 150 °C in an oxygen-free environment for 30 min. Then, they were heated up to 300 °C in an anaerobic environment and the reaction was continued for 30 min. Next, the obtained solution was centrifuged at 10,000 rpm for 5 min and the precipitation was gathered and washed with ethanol. Washing was repeated until the supernatant became colorless, resulting in nanomagnetic Fe_3_O_4_.

Nanomagnetic Fe_3_O_4_ (8 mg) and PLGA (50 mg) were added to 5 ml of dichloromethane (DCM) and thoroughly stirred to dissolve. Then, the dissolved solution was added to a pre-prepared 20-ml 0.3% PVA aqueous solution. The layered mixed solution was sonicated for 10 min to fully emulsify, and DCM was removed by rotary evaporation. The mixture was left to stand for 5 min. All fluids were taken and centrifuged for 10 min at 10,000 rpm. Then, the sediments were collected and washed with deionized water until the supernatant became colorless, resulting in Fe_3_O_4_@PLGA-PVA (FP).

DOX (16 mg) was added to the raw materials for FP synthesis. The layered mixed solution was sonicated for 10 min to fully emulsify, and DCM was removed by rotary evaporation. The mixture was left to stand for 5 min. All fluids were taken and centrifuged for 10 min at 10,000 rpm. Then, the sediments were collected and washed with deionized water until the supernatant became colorless, resulting in FDP. After testing, the drug loading rate of DOX is 25%.

The freeze-dried CMs were added to FDP solution with a mass ratio of FDP:CM = 1:2, ensuring that an excess of CM enveloped FDP. Then, ultrasound was used to shake for 10 min. The mixture was centrifuged at 10,000 rpm for 5 min, and the precipitate was resuspended in equal volume. Finally, a nanomaterial extruder was used to extrude and obtain FDPM.

### Material characterizations

Observation of field-emission scanning electron microscopy (SEM) and energy-dispersive X-ray (EDX) pattern analysis were performed on the SU8100 electron microscope (Hitachi, Japan). Transmission electron microscopy (TEM) was used for analysis on HT7800/HT7700 electron microscopy (Hitachi, Japan). The fluorescence spectra were performed on RF-6000 (Shimadzu, Japan). X-ray photoelectron spectroscopy (XPS) was performed on Escalab 250Xi (Thermo Fisher Scientific, USA). X-ray diffraction (XRD) analysis was performed on the D8 Advance (Bruker, Germany) diffractometer. Fourier transform infrared (FTIR) spectroscopy was recorded on the Nicolet iS50 spectrometer (Thermo Fisher Scientific, USA) in the range of 400 to 4,000 cm^−1^. Zeta potential and particle size analysis were performed on the Zetaview (PMX120-Z) nanoparticle tracking analyzer (Particle Metrix, Germany). The protein composition of FDPM surface and CM was analyzed by sodium dodecyl sulfate–polyacrylamide gel electrophoresis (SDS-PAGE).

### Cell culture

GL261 cells, U87 cells, human umbilical vein endothelial cells (HUVECs), and A549 cells were obtained from Shanghai Zhong Qiao Xin Zhou Biotechnology Co. Ltd. and cultivated in complete Dulbecco’s modified Eagle’s medium (DMEM) containing 10% fetal bovine serum (FBS; GIBCO), 1% streptomycin sulfate, and 1% penicillin G sodium. Cells were incubated in a humid atmosphere of 37 °C, 95% air, and 5% CO_2_. Cultivation, amplification, cryopreservation, and resuscitation were all carried out according to the recommended cell culture protocol.

GSCs were obtained through GL261 cell-induced sorting. Pre-frozen GL261 cells were revived and cultured in DMEM/F12 (1:1, v/v) medium, which contained 1% penicillin G sodium and 1% streptomycin sulfate, with the addition of 1:50 B27, 20 ng ml^−1^ epidermal growth factor, and 20 ng ml^−1^ basic fibroblast growth factor. Cells were incubated at 37 °C in a 95% air and 5% CO_2_ humidified atmosphere. Fresh culture medium containing the aforementioned cytokines was added every 3 to 4 d and passaged once every 7 to 9 d. When the cells form suspended cell spheres, it indicated that they had grown like stem cells. When cultured for more than 3 generations, the cell spheres were digested with Accutase (Sigma-Aldrich) and dispersed into a single-cell suspension. The cells were incubated with CDl33 antibodies, and CD133-positive cells were selected by a BD FACSAria Fusion flow cytometry sorter (BD, USA) for further cultivation.

### Extraction and purification of CM and CM proteins

Cells (2 × 10^7^ to 5 × 10^7^) were collected, washed 3 times with precooled phosphate-buffered saline (PBS) in an appropriate amount of ice bath, and centrifuged at 4 °C and 600*g* for 5 min to precipitate them. After discarding the supernatant at 4 °C, the cell precipitate was further centrifuged at 600*g* for 1 min to get rid of any remaining liquid. Benzenesulfonyl fluoride (10 μl) (Beyotime Biotechnology, China) was added to 1 ml of membrane protein extraction reagent A (Beyotime Biotechnology, China) to achieve a final concentration of 1 mM. After mixing well, it was added to washed cells and completely resuspended. The solution was then placed in an ice bath for 15 min. Then, the cells were crushed under an ultrasonic crusher for 5 s and centrifuged at 4 °C and 700*g* for 10 min, and the supernatant was collected to further centrifuge at 4 °C and 14,000*g* for 30 min to precipitate membrane fragments. Next, a reasonable amount of TE buffer was added to the collected precipitate and washed for 3 min to dissolve any remaining nucleic acids. The solution was centrifuged at 14,000*g* for 30 min at 4 °C to obtain purified CM. The obtained CMs were freeze-dried, weighed, and stored at −80 °C for future use. Some of the obtained CM was centrifuged at 14,000*g* for 10 s at 4 °C, and the supernatant was completely absorbed. Membrane protein extraction reagent B (200 μl) (Beyotime Biotechnology, China) was added to the above precipitate, and the precipitate was resuspended with Vortex at the highest speed and intensity for 5 s, followed by an ice bath for 10 min. The ice bath incubation and Vortex steps needed to be repeated at least twice. Subsequently, the mixture was centrifuged at 14,000*g* for 10 min at 4 °C, and the supernatant, i.e., cell membrane proteins, was collected and stored at −80 °C for later use.

### Cellular uptake

#### Uptake of different materials by GSCs

GSCs were digested to obtain a single-cell suspension and then seeded into the wells of 6-well plates (2 × 10^5^ cells per well) (Nest Biotech, China). After culturing for 6 h, the new medium was changed, and FDP and FDPM were added (standardized with 10 μg ml^−1^ DOX). Four hours later, the plates with cells were washed 3 times with PBS and then analyzed on BD LSRFortessa (BD, USA).

The 24-well plates were prepared and wrapped overnight with poly-l-lysine (PLL) (Sigma-Aldrich), and then the plates were washed 3 times with PBS, each time for 5 min. GSCs were digested to obtain a single-cell suspension, which was seeded into wells of the 24-well plates (8 × 10^4^ cells per well). After 24 h of culturing, the medium was changed, and FDP and FDPM were added (standardized with 10 μg ml^−1^ DOX). Four hours later, the plates with cells were washed 3 times and then subjected to fluorescence analysis using a Thunder microscope (Leica, Germany).

#### Uptake of FDPM or FDPM^GL261^ by different tumor cells

GSCs, GL261, U87, and A549 cells were digested and resuspended to obtain cell suspensions, which were seeded into wells of 6-well plates (2 × 10^5^ cells per well). After 6 h of culturing, the medium was changed, and FDP and FDPM were added (standardized with 10 μg ml^−1^ DOX). Four hours later, the plates with cells were washed 3 times and then analyzed on BD LSRFortessa (BD, USA).

### In vitro cytotoxicity test

The 96-well plates were coated overnight with PLL, and the plates were washed 3 times with PBS for 5 min each time. GSC suspension was inoculated into the 96-well plates with 0.80 × 10^4^ cells per well. For both FDP and FDPM groups, 100 μl of stem cell culture medium was added to each well at the concentrations of FDP or FDPM (standardized with 5, 10, 15, 20, 40, 60, 80, and 100 μg ml^−1^ FDP). After 24 h of incubation, the fresh culture medium was replaced and the CCK-8 assay kit was used (Beyotime Biotechnology, China) to detect cell activity.

### Measurement of cell sphere permeability of FDP and FDPM

The confocal special glass bottom culture dish (BioSharp, China) was coated overnight with PLL, and after the coating was completed, it was washed 3 times with PBS for 5 min each time. Uniformly sized GSC spheres were inoculated into 24-well plates, and FDP or FDPM was added to each well (standardized with 10 μg ml^−1^ DOX). After incubation for 4 h, the cell spheres were washed 3 times and transferred to the prepared glass bottom culture dish for observation using a confocal microscope (Zeiss, Germany).

### In vitro measurement of BBB penetration

The establishment of an in vitro BBB model utilized the Transwell culture method (LABSELECT, China). The lower chamber of the Transwell apparatus was coated overnight with PLL and then washed 3 times with PBS, each wash lasting 5 min. BEnd.3 cells were cultured in the upper chamber of the Transwell apparatus, and the cells were interconnected to form tight junctions. When a Millicell ERS voltmeter was used to measure the transendothelial resistance between the upper and lower compartments exceeding 200 Ω cm^−2^, it indicated that the model had been successfully established. GSCs were digested to obtain a single-cell suspension and seeded into the lower chamber of the Transwell (8 × 10^4^ cells per well). After culturing for 24 h, the fresh culture medium was changed. FDP or FDPM (standardized with 100 μg ml^−1^ DOX) was then added to the upper chamber of the Transwell. After a further 24-h incubation, the cells were washed 3 times with PBS and then analyzed using a BD LSRFortessa for flow cytometry. Fluorescence analysis was performed using a Thunder microscope (Leica, Germany). Apoptosis detection was carried out using a TUNEL (terminal deoxynucleotidyl transferase mediated deoxyuridine triphosphate nick end labeling) staining kit (Meilunbio, China).

### Growth changes of GSC spheres after BBB penetration

As previously described, the BBB model was established using the Transwell culture method. Uniformly sized GSC spheres were seeded into the lower chamber of the Transwell (without PLL coating). FDP or FDPM (standardized with 100 μg ml^−1^ DOX) was added to the upper chamber of the Transwell. The culture medium was replaced every 2 d, and the growth of the GSC spheres was observed under a Thunder microscope (Leica, Germany) on days 1, 3, 5, and 7.

### In vitro evaluation of hemolysis

The blood samples of BALB/c nude mice were collected in anticoagulant-treated Eppendorf tubes using the enucleation method. The collected samples were then centrifuged at 3,000 rpm for 5 min and washed 3 times with PBS to attain red blood cells (RBCs). The collected RBCs were diluted 50 times. FDPM (100 μl; 80 μg ml^−1^) was added to 100 μl of suspended RBCs and hatched at 37 °C for 2 h. After incubation, it was centrifuged at 10,000 rpm for 10 min. To evaluate hemolytic activity, the released hemoglobin in the sample was measured by measuring the absorbance (*A*) at 570 nm. The PBS treatment group was used as a negative control, while the deionized water treatment group was used as a positive control. The percentage of hemolysis was calculated as $\begin{array}{c} {}[(A_{\text{sample}}-A_{\text{negative control}})/(A_{\text{positive control}}-A_{\text{negative control}})]\times 100 \end{array}$.

### Experimental animals

BALB/c nude mice (18 to 22 g, 6 to 8 weeks old) were provided by the Experimental Animal Center of Nantong University of China. All animals were raised in an environment with suitable temperature and humidity, able to freely access water and food. Mice experienced 12-h light/12-h dark cycle every day. All animal experiments used in this study were conducted in accordance with the institutional guidelines for animal care and use. This research protocol was approved by the Care and Use Committee of Laboratory Animal Research Center of Nantong University (ethical approval number: S20240116-009).

### Establishment of an animal model for evaluating antitumor efficacy

The nude mice were anesthetized with 1 to 5% isoflurane (RWD, China) mixed with oxygen. The mice were fixed using a mouse brain stereotactic injection device (RWD, China). After head disinfection, GSCs (1 × 10^4^ cells per mouse, injection volume 2 μl) were slowly injected into the right side of the mouse brain (lateral 2.0 mm, anterior 0.5 mm, depth 3 mm) at a rate of 0.5 μl min^−1^. After injection was completed, the needle was used to seal the injection point for 1 min, then it was slowly removed, and the skin was disinfected again and sutured.

To evaluate the distribution of intravenously injected drugs in tumor-bearing nude mice, Cy7-labeled FDP was prepared. Briefly, Cy7 was slowly added to 1 ml of FDP suspension and magnetically stirred in the dark at room temperature for 48 h. After stirring, the mixed solution was centrifuged at 10,000 rpm for 5 min and washed 3 times with deionized water to remove unbound Cy7. ^Cy7-^FDP was obtained and then used to prepare FDPM (^Cy7-^FDPM). On day 12 after model establishment, tumor-bearing nude mice were randomly divided into 4 groups: ^Cy7-^FDP group, ^Cy7-^FDP + magnet group, ^Cy7-^FDPM group, and ^Cy7-^FDPM + magnet group. ^Cy7-^FDP or ^Cy7-^FDPM was administered intravenously (standardized with 10 mg kg^−1^ DOX). The FDP + magnet group and FDPM + magnet group received magnetic field guidance for 30 min after injection. The distribution of the drug was monitored at 1, 2, 4 and 8 h using a small-animal live imaging system (IVIS Spectrum, USA). At 4 h, mice were euthanized and organs were collected for drug distribution analysis.

The tumor-bearing nude mice were stochastically divided into 6 groups: control group, FP group, FDP group, FDPM group, FDPM + magnet group, and temozolomide (TMZ) group. From day 12 to day 16, FP, FDP, or FDPM was administered intravenously (standardized with 20 mg kg^−1^ FDPM). For the TMZ group, 50 mg kg^−1^ TMZ was administered by gavage once a day from day 12 to day 16. The FDPM + magnet group received magnetic field guidance for 30 min after injection. Six mice were raised in each group to monitor their survival status. Body weight was measured every 3 d, and the final survival time of the tumor-bearing nude mice was recorded.

To obtain luciferase-expressing GSCs (^luc-^GSCs), GSCs were transfected with a luciferase lentivirus purchased from Shanghai Genechem Co. Ltd. The tumor-bearing nude mice model was established using stable ^luc-^GSCs (1 × 10^4^ cells per mouse). The tumor-bearing nude mice were stochastically divided into 5 groups: control group, FP group, FDP group, FDPM group, and FDPM + magnet group. Each group contained 6 mice. From day 12 to day 16, FP, FDP, or FDPM was administered intravenously (standardized with 20 mg kg^−1^ FDPM). The FDPM + magnet group received magnetic field guidance for 30 min after injection. On days 12, 15, 18, and 21, d-luciferin salt (150 mg kg^−1^) was injected intravenously, and tumor growth was monitored using a small-animal live imaging system. On day 21, mice were euthanized and brain tissues were subjected to hematoxylin and eosin (H&E) staining and TUNEL staining.

### In vivo biosafety assessment

BALB/c nude mice were intravenously injected with FDPM at a dose of 20 mg kg^−1^ for 6 consecutive days. Fourteen days after the injection, the mice were euthanized, and routine blood tests and serological markers were collected. The major organs, including the heart, liver, spleen, lungs, and kidneys, were harvested for H&E staining.

### Statistical analysis

Each experiment in this study was independently repeated at least 3 times. All data were expressed as mean ± SD. The statistical analysis of the 2 groups was conducted using a Student’s *t* test, whereas the analysis among multiple groups was evaluated using one-way analysis of variance. The survival data were determined using the Kaplan–Meier method and compared using the log-rank (Mantel–Cox) test. *P* value was calculated using GraphPad Prism 8.0 statistical software. Significant differences are indicated as n.s. *P* > 0.05, *P < 0.05, **P < 0.01, ***P < 0.001, and ****P < 0.0001.

## Results and Discussion

### Characterization of nanoparticles

**Fig. 2.**
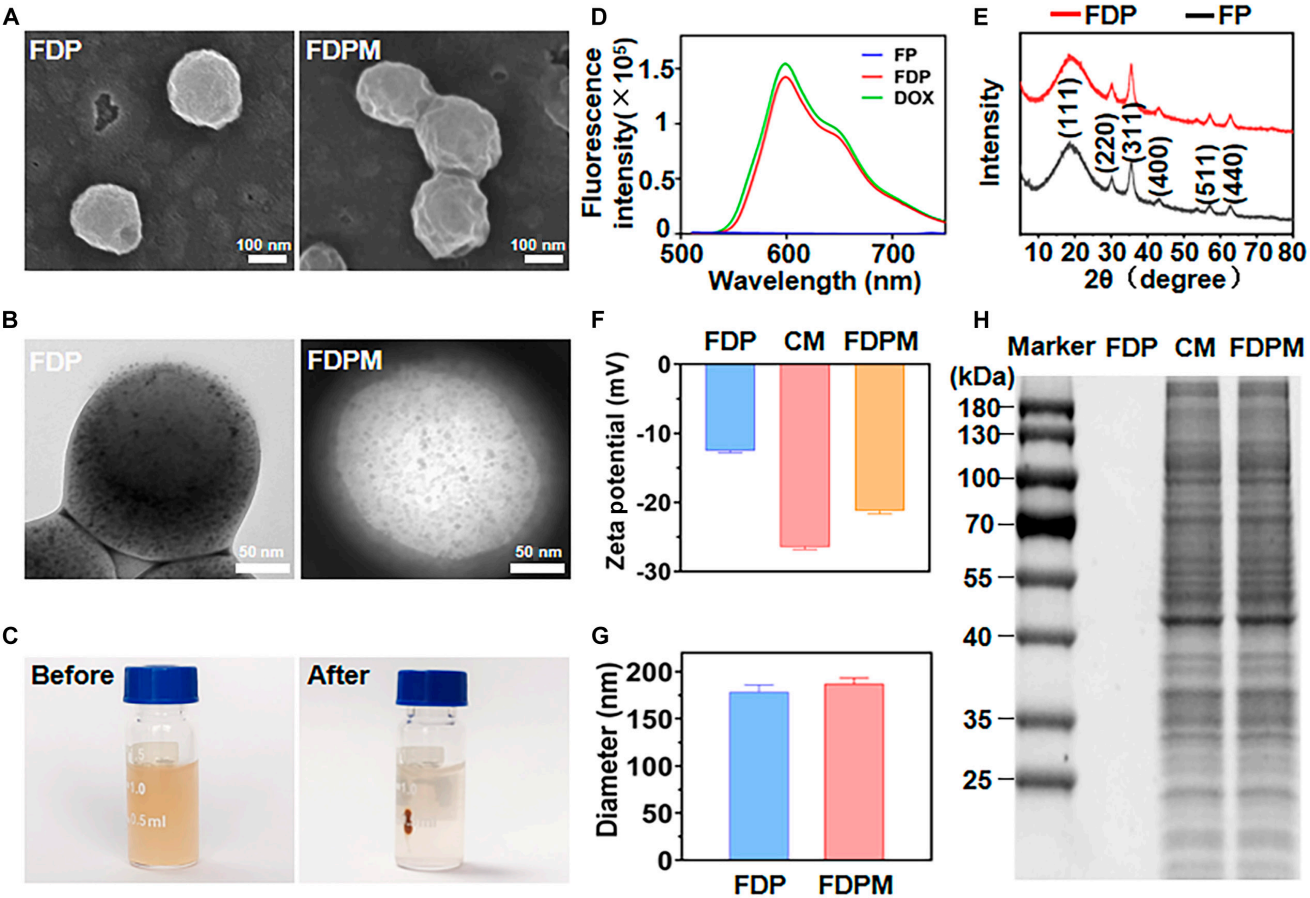
Structural and compositional characteristics of FDP and FDPM. Representative SEM (scale bar, 100 nm) (A) and TEM (scale bar, 50 nm) (B) images of FDP and FDPM. (C) Magnetic field response capability of FDP. (D) Fluorescence spectra of FP, FDP, and DOX. (E) XRD patterns of FDP and FP. (F) Zeta potentials of FDP, CM, and FDPM. (G) Diameter of FDP and FDPM. (H) SDS-PAGE protein analysis of FDP, CM, and FDPM. Data are shown as mean ± SD (*n* = 3).

We utilized an emulsion solvent evaporation method to prepare magnetic nanoparticle FDP and obtained FDPM through low-temperature ultrasonic assistance and a nanomaterial extruder. As shown in Fig. 2A, SEM results indicated that FDP appeared as spherical particles with a rough surface.

After coating with CM, the particles exhibited typical membrane-like folds, indicating that CM successfully encapsulated the surface of FDP and produced FDPM. EDX spectroscopy analysis (Fig. S1) revealed successful loading of iron (Fe) and nitrogen (N) elements in FDP, indicating the successful integration of Fe_3_O_4_ and DOX into the nanosystem. TEM results in Fig. 2B showed small Fe_3_O_4_ particles distributed within the PLGA and PVA framework. After CM encapsulation, a thin membrane-like layer covered the surface of the nanoparticles. Our institution’s self-developed magnetic field generator (Fig. S2) was used to further examine the successful loading of Fe_3_O_4_ particles. Figure 2C demonstrates that after 30 min of magnetic field guidance, the original uniformly distributed nanoparticles almost congregated at the magnetic attraction point, and the CM coating did not affect the material’s magnetic responsiveness (Fig. S3).

The fluorescence spectra of FP, FDP, and DOX are shown in Fig. 2D. There was no fluorescence signal in FP, but FDP and DOX had almost identical fluorescence signals, indicating the successful loading of DOX in FDP. Subsequently, XRD in Fig. 2E showed no significant differences between the diffraction spectra of FP and FDP, both supporting characteristic diffraction peaks of iron oxides. XPS analysis compared the spectra of FP and FDP (Fig. S4). The results revealed the presence of N1s XPS signals in FDP, distinct from FP, while the Fe2p3 XPS signals showed no significant difference between FP and FDP, indicating successful loading of DOX in FDP. Interestingly, FTIR showed almost identical spectra for FP and FDP, due to the incorporation of DOX, FDP exhibited a more pronounced transmission ratio (Fig. S5). Combining XPS, FTIR, and EDS (energy-dispersive spectrometer) analyses, the presence of C and N signals suggested residual stabilizers of PVA in the materials. However, the stronger N signals in FDP could be attributed to the presence of DOX.

The drug loading percentage of DOX in FDP was then determined. First, the absorbance of DOX at 488 nm was measured, establishing a standard curve for DOX (Fig. S6). After synthesizing FDP and completing washing and centrifugation, the absorbance of all supernatants at 488 nm was measured, calculating the mass of DOX in the supernatant (*M*_*DOX supernatant*_). Knowing the total mass of DOX (*M*_*DOX total*_), the drug loading percentage of FDP was calculated as (*M*_*DOX total*_ *− M*_*DOX supernatant*_)*/M*_*FDP*_ *× 100%*, resulting in a loading rate of 25%. Additionally, the prepared FDP dispersed well in various solvents such as simulated body fluid (SBF), PBS, and deionized water, indicating good stability in these media (Fig. S7). We further tested the drug release of FDPM in SBF by dividing 40 μg ml^−1^ FDPM into 10 groups (*n* = 3) and centrifuging at 0, 8, 16, 24, 32, 40, 48, 56, 64, and 72 h. The drug cumulative release percentage of FDPM was calculated as *M*_*DOX supernatant*_/*M*_*DOX loading*_ *× 100%* (Fig. S8).

The surface zeta potential of FDP, CM, and FDPM was measured to further confirm the CM coating. After CM encapsulation, the zeta potential of FDPM changed from −12.54 to −21.21 mV, approaching the surface zeta potential of CM at −26.46 mV (Fig. 2F). Figure 2G measures the particle diameter of FDP before and after CM coating, where the average size of the nanostructure increased from 178 to 186 nm after CM coating, indicating a coating thickness of approximately 8 nm. Figure S9 detailed the particle size distribution of FDP and FDPM.

The protein analysis using SDS-PAGE also demonstrated the presence of proteins in FDPM that were equivalent in content to purified CM, in stark contrast to the situation where there were almost no proteins present in FDP (Fig. 2H). In summary, these properties of FDP and FDPM laid the foundation for their application in the biological field.

### Culture and identification of GSCs

**Fig. 3.**
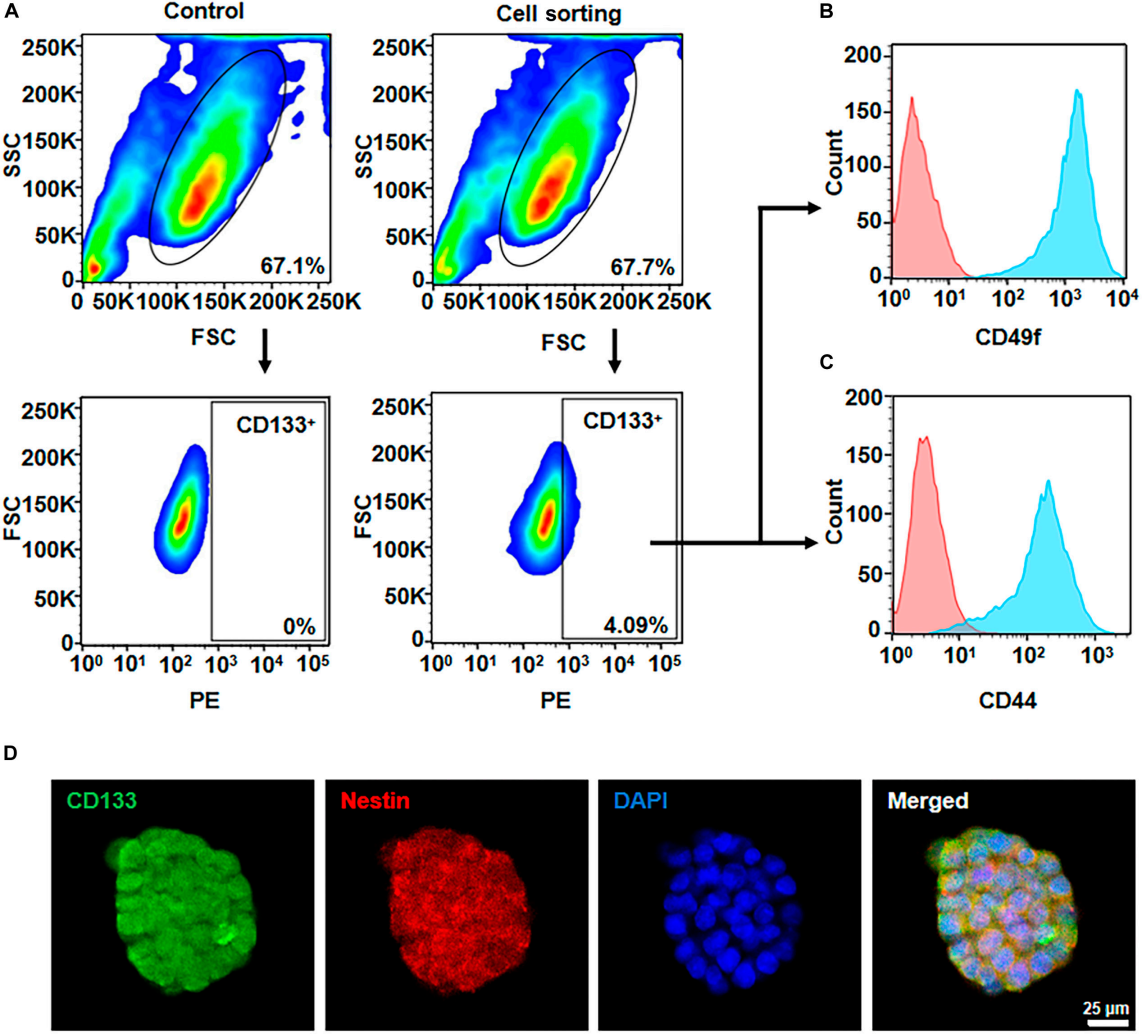
Sorting and identification of GSCs. (A) Flow cytometry sorting strategy for GSCs. Flow cytometry detection of characteristic molecules including CD49f (B) and CD44 (C) in GSCs. (D) Fluorescence detection of characteristic markers in GSCs. Scale bar, 25 μm.

GSCs were obtained using a reverse separation method. When cells grew in suspended spheres, it indicates that they have stem cell characteristics (Fig. S10). The flow sorting strategy is shown in Fig. 3A. The sorted CD133^+^ cells constituted 4.09% of the total cell population.

After expansion and stabilization of these sorted CD133^+^ cells, their stem cell properties were further verified [35]. Initially, flow cytometry identification using CD49f and CD44, 2 characteristic surface molecules, showed high expression of these molecules in CD133^+^ cells (Fig. 3B and C) [36,37]. Subsequently, the expression of CD133, Nestin, and Sox2 in cell spheres was analyzed. Immunofluorescence analysis revealed (Fig. 3D and Fig. S11) that the cell spheres expressed these typical stem cell markers CD133, Nestin, and Sox2 [38,39]. Interestingly, it was questioned whether GL261 cells inherently express CD133 and Nestin. An examination of these characteristic molecules in GL261 cells (Fig. S12) showed that GL261 cells were negative for both CD133 and Nestin. This confirmed the successful induction of GSCs.

Further, a protein analysis of the cell membrane proteins of GL261 cells and GSCs was conducted using SDS-PAGE. The results demonstrated that GSCs contained all the cell membrane protein bands of GL261 cells, and in some bands, the protein content in GSCs was markedly higher than that in GL261 cells, as indicated by frames a and c. Notably, GSCs even showed new protein bands in certain areas, as marked by frame b (Fig. S13). Thus, differences in the composition of cell membrane proteins between GL261 cells and GSCs were observed, which may relate to the unique biological functions of GSCs compared to regular tumor cells and warrant further in-depth research.

### Cellular uptake and toxicity of FDPM in vitro

**Fig. 4.**
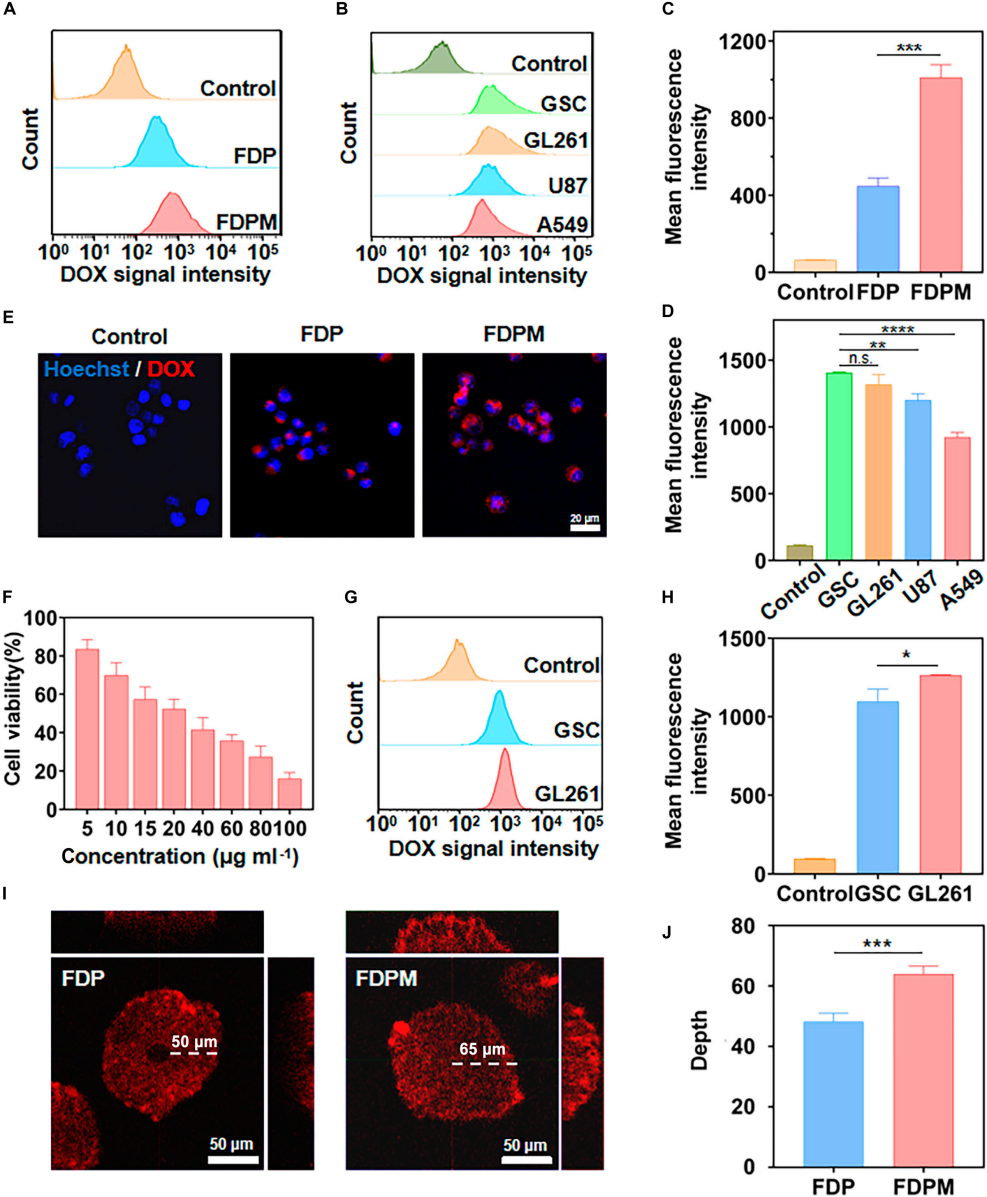
Cellular uptake and toxicity of FDPM. Flow cytometry analysis of cellular uptake with different nanoparticles (A) or within different cells (B). (C and D) Corresponding quantification of (A) and (B). Data are expressed as mean ± SD (*n* = 3). **P < 0.01, ***P < 0.001, ****P < 0.0001. (E) Fluorescence photos of GSCs’ uptake of FDP and FDPM (standardized with 10 μg ml^−1^ DOX for 4 h). Scale bar, 20 μm. (F) Relative survival rate of GSCs after 24 h of treatment with different concentrations of FDPM. Data are expressed as mean ± SD (*n* = 4). (G) Flow cytometry analysis of GSCs or GL261 cells’ uptake of FDPM^GL261^ (coated with GL261 cell membranes) (40 μg ml^−1^ for 4 h). (H) Quantification of average intracellular fluorescence intensity. Data are expressed as mean ± SD (*n* = 3). *P < 0.05. (I) Depth of uptake and penetration of FDP and FDPM (standardized with 10 μg ml^−1^ DOX for 4 h) by GSC cell spheres. Scale bar, 50 μm. (J) Corresponding quantification of (I). Data are expressed as mean ± SD (*n* = 3). ***P < 0.001.

To evaluate whether the uptake of nanoparticles by target cells differed before and after CM modification, GSCs were divided into 3 groups and each received PBS, FDP, or FDPM treatment (standardized with 10 μg ml^−1^ DOX). After 4 h of treatment, cells were collected and the fluorescence intensity of DOX was measured using a flow cytometer. The results, as shown in Fig. 4A and C, indicated that compared to the control group, GSCs significantly took both FDP and FDPM. However, FDPM, coated with CM, was more readily internalized by GSCs (*P* < 0.001), evidently enhancing the enrichment of nanoparticles within the target cells.

For a more direct observation of this ability of FDPM, GSCs were seeded to PLL-coated wells to ensure firm attachment and growth. Similar to the previous groups, cells were treated with FDP or FDPM (standardized with 10 μg ml^−1^ DOX) for 4 h. Fluorescence microscopy observations, as depicted in Fig. 4E, similarly showed that FDPM was more readily internalized by GSCs.

Considering the enhanced uptake of FDPM by GSCs, we explored how different cell types would internalize FDPM. Four different tumor cell lines including GSCs, GL261, U87, and A549 were treated with FDPM (40 μg ml^−1^) for 4 h, and flow cytometry was used to measure the fluorescence intensity of DOX within various cells again. As shown in Fig. 4B and D, GSCs and GL261 cells exhibited efficient uptake of FDPM, while the human-derived glioma line U87 cells showed weaker uptake than the former 2 (*P* < 0.01). The nonglioma cell line A549 cells had the lowest ability to internalize FDPM (*P* < 0.0001). This demonstrated that FDPM, coated with CM, has significant homologous targeting ability toward GSCs, and since the GSCs were induced from GL261 cells, FDPM also showed homologous targeting ability toward GL261 cells. This dual-targeting capability of FDPM enhances our confidence in its potential for targeting and killing tumor cells at the tumor site.

Considering the targeting ability of CM, it was hypothesized that GL261 cell membranes might also possess this ability. Thus, FDPM^GL261^ was prepared using GL261 cell membranes for comparative validation. As shown in Fig. 4G and H, the uptake of FDPM^GL261^ by GL261 cells was significantly stronger than by GSCs (*P* < 0.05). Moreover, by further comparing the uptake effects of GSCs on FDPM or FDPM^GL261^ (Fig. S14), it was found that FDPM^GL261^ had a much lower targeting effect on GSCs than FDPM (*P* < 0.01). Therefore, it could be concluded that CMs possess the ability to target both GSCs and GL261 cells, whereas GL261 cell membranes do not target GSCs.

We further observed the difference in FDPM uptake between GSCs and HUVECs when cocultured, as shown in Fig. S15. When green fluorescent protein (GFP)-labeled HUVECs and GSCs coexisted, due to the homologous targeting effect of FDPM, GSCs could uptake a large amount of FDPM. Compared to GSCs, HUVECs had almost no uptake of FDPM, indicating a higher degree of homologous targeting of FDPM and suggesting that FDPM had less impact on normal tissue cells.

Cell cytotoxicity tests were conducted for FDPM. As shown in Fig. 4F, the cytotoxicity of FDPM increased with increasing dosage, with approximately 60% cell death at the concentration of around 40 μg ml^−1^. Since GSCs typically grow as floating cell spheres, this characteristic was utilized to observe the penetration ability of FDPM into the deeper parts of the cell spheres. Figure 4I and J demonstrates that after 4 h of treatment with either FDP or FDPM (standardized with 10 μg ml^−1^ DOX), FDP reached about 50 μm deep into the cell sphere through stepwise uptake by GSCs. In comparison, FDPM penetrated to a depth of at least 65 μm.

As could be seen from the above, FDPM not only was more readily internalized by cells but also simultaneously targeted GSCs and GL261 cells, demonstrating a stronger ability to reach deeper sites. This provided robust support for its potential in vivo applications.

### FDPM’s ability to penetrate the BBB and kill tumors

**Fig. 5.**
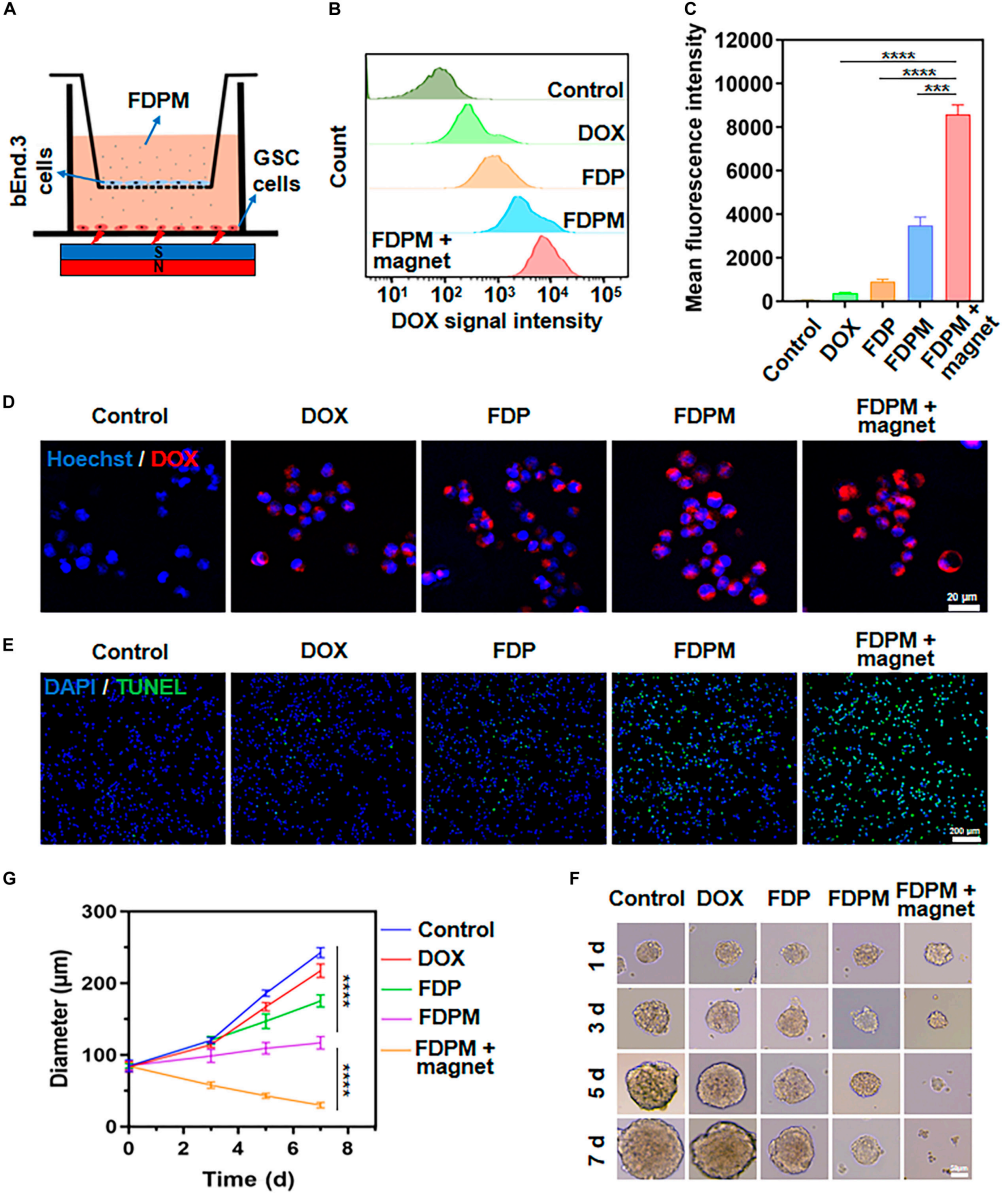
Evaluation of BBB penetration performance and cell killing effect after penetration. (A) Schematic diagram of BBB permeation model using the Transwell system. (B) Flow cytometry analysis of different experimental groups passing through the BBB (standardized with 100 μg ml^−1^ DOX). (C) Corresponding quantification of the average fluorescence intensity of GSCs. Data are expressed as mean ± SD (*n* = 3). ***P < 0.001, ****P < 0.0001. (D) Fluorescence images of GSCs taken up by different experimental groups (standardized with 100 μg ml^−1^ DOX) passing through the BBB. Scale bar, 20 μm. (E) TUNEL staining of GSCs treated with different experimental groups (standardized with 100 μg ml^−1^ DOX) after passing through the BBB. Scale bar, 200 μm. The growth changes (F) and corresponding quantification (G) of GSC cell spheres in each experimental group (standardized with 100 μg ml^−1^ DOX) in the BBB model. Data are expressed as mean ± SD (*n* = 4). ****P < 0.0001. Scale bar, 50 μm.

It is well known that the presence of the BBB is a major obstacle in the treatment of gliomas. Given the clear targeting capability of FDPM, whether FDPM facilitates the passage of nanoparticles through the BBB warrants investigation. Figure 5A illustrates our constructed in vitro BBB model using a Transwell apparatus. After forming tight junctions by bEnd.3 cells in the upper chamber of the Transwell and passing the transendothelial electrical resistance test, the corresponding nanoparticles were added to the upper chamber of the Transwell. For groups requiring magnetic guidance, magnets were placed at the bottom of the apparatus.

The uptake of nanoparticles by the cells was analyzed using the intrinsic fluorescence of DOX, as shown in Fig. 5B and C. Pure DOX (100 μg ml^−1^) was ineffective in penetrating the BBB for cellular uptake. Although FDP (standardized with 100 μg ml^−1^ DOX) showed improved efficiency in crossing the BBB compared to DOX, its efficiency was still much lower than that of FDPM (standardized with 100 μg ml^−1^ DOX). With magnetic field guidance, FDPM easily penetrated the BBB and was internalized by GSCs (*P* < 0.0001). The fluorescence analysis in Fig. 5D also confirmed the high efficiency of FDPM in crossing the BBB under magnetic field guidance.

Given the efficient penetration of FDPM through the BBB with magnetic field assistance, we further verified whether the nanoparticles could induce effective cell apoptosis after passing through the BBB in the Transwell apparatus. As shown in Fig. 5E, pure DOX or FDP was almost ineffective in killing GSCs. FDPM had some cytotoxic effects, but they were not pronounced. However, with magnetic field guidance, FDPM significantly improved in efficiency, almost completely killing all GSCs.

Subsequently, to evaluate whether the nanoparticle’s ability to kill cell spheres remained effective, uniformly sized GSC cell spheres were directly seeded in the lower chamber of a Transwell apparatus without PLL coating. Over several days of observation, as depicted in Fig. 5F and G, FDPM with magnetic field guidance significantly inhibited the growth of the cell spheres (*P* < 0.0001). The GSC cell spheres gradually shrank and disintegrated.

Overall, these findings suggested that FDPM, especially under magnetic field guidance, effectively crossed the BBB and had a significant impact on inhibiting the growth and inducing apoptosis of glioma cells, demonstrating its potential as a therapeutic agent in the treatment of gliomas.

### Targeting effects of FDPM in vivo

Numerous studies have indicated that the presence of GSCs contributes to drug resistance, radiation resistance, and postsurgical recurrence in gliomas. Therefore, we established an in situ glioma model in nude mice by injecting GSCs into their brains to observe the median survival time of the mice (Fig. S16). When 1 × 10^5^ GSCs per mouse were used, the mice died approximately 7 d after injection. With 5 × 10^4^ GSCs per mouse, the median survival period was extended to about 12 d. In comparison, using standard tumor cells like 5 × 10^5^ GL261 cells per mouse could achieve a median survival of about 31 d, whereas only 1 × 10^4^ GSCs per mouse were needed for a similar median survival of 26 d. This indicated that GSCs are more malignant and have a stronger tumor-forming ability compared to standard tumor cells.

**Fig. 6.**
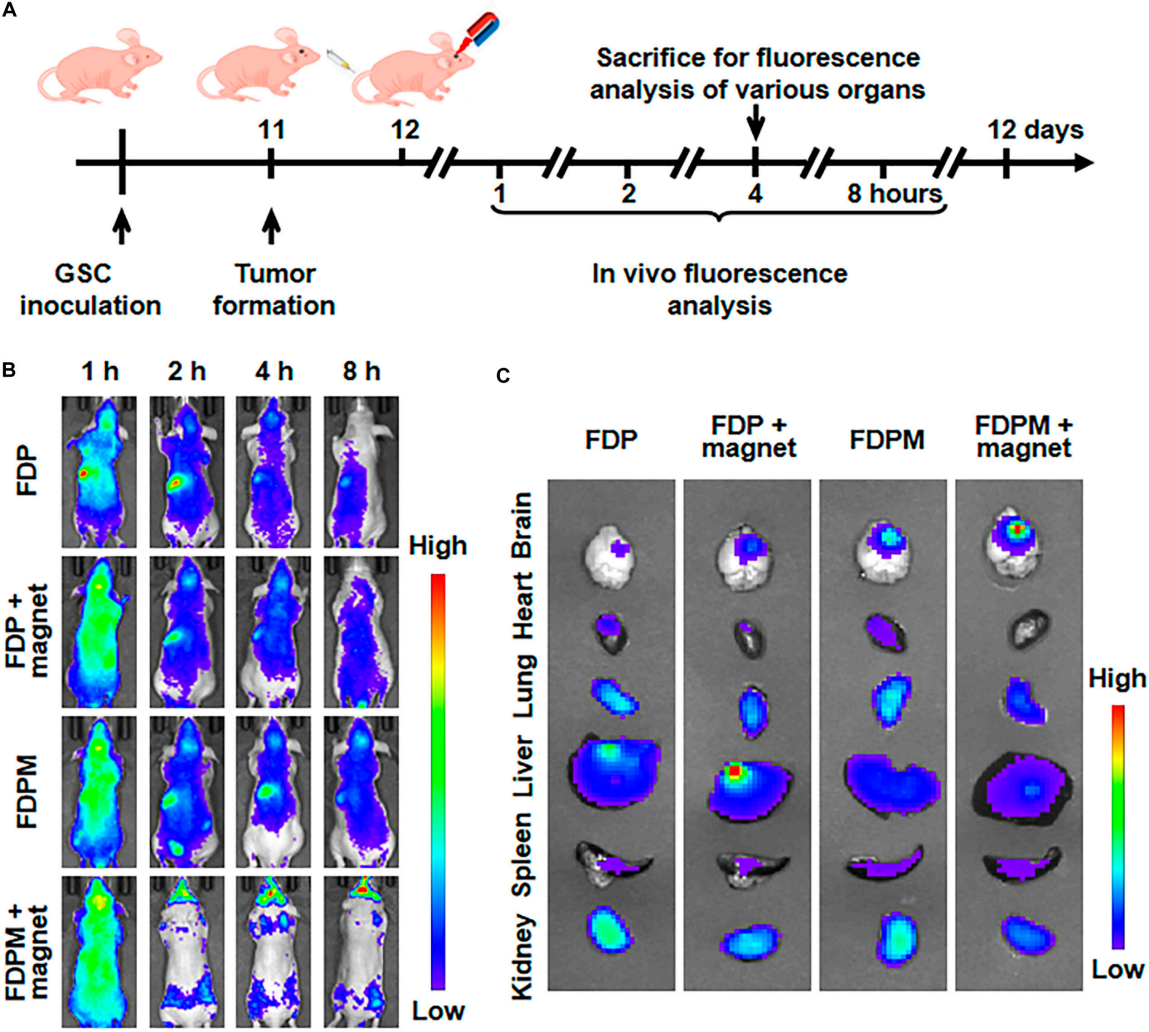
In vivo targeting effects. (A) Schematic illustration of the targeted experimental design. (B) Representative in vivo fluorescence images of tumor-bearing nude mice at indicated time points (1, 2, 4, and 8 h) after intravenous injection of FDP or FDPM (standardized with 10 mg kg^−1^ DOX) (*n* = 3). (C) Ex vivo images of various organs examined at 4 h after injection (standardized with 10 mg kg^−1^ DOX) (*n* = 3).

Therefore, we constructed an in situ glioma model in nude mice using 1 × 10^4^ GSCs per mouse. We then applied FDPM combined with magnetic field guidance (30 min of guidance after each administration), as shown in the basic procedure in Fig. 6A. To assess the in vivo targeting capability of FDPM, we injected cy7-labeled FDP or FDPM (FDP^cy7^ or FDPM^cy7^) (standardized with 10 mg kg^−1^ DOX) into the mice via tail vein injection after successfully establishing the in situ glioma model. Using a small-animal live imaging system, we observed the distribution of nanoparticles in the body as shown in Fig. 6B. With the passage of time, the distribution and accumulation of nanosystems in the body gradually changed, up to about 8 h. FDP^cy7^ was almost not present at the tumor site and was largely metabolized by the body, whereas FDPM^cy7^ still showed marked retention in the body and did not undergo extensive metabolism. Moreover, its accumulation at the tumor site gradually increased, and this effect was further enhanced with magnetic field guidance. Four hours after administration of nanoparticles, mice were euthanized, and the distribution of nanoparticles in various organs was analyzed. Figure 6C shows that without magnetic field guidance, FDP^cy7^ tended to accumulate in the liver and kidney, while FDPM^cy7^ was more inclined to accumulate in the tumor site. Under magnetic field guidance, the accumulation of FDP^cy7^ in the kidney or liver decreased, with some movement toward the head, while FDPM^cy7^ clearly congregated at the tumor site.

### The ability of FDPM to inhibit tumor growth in vivo

**Fig. 7.**
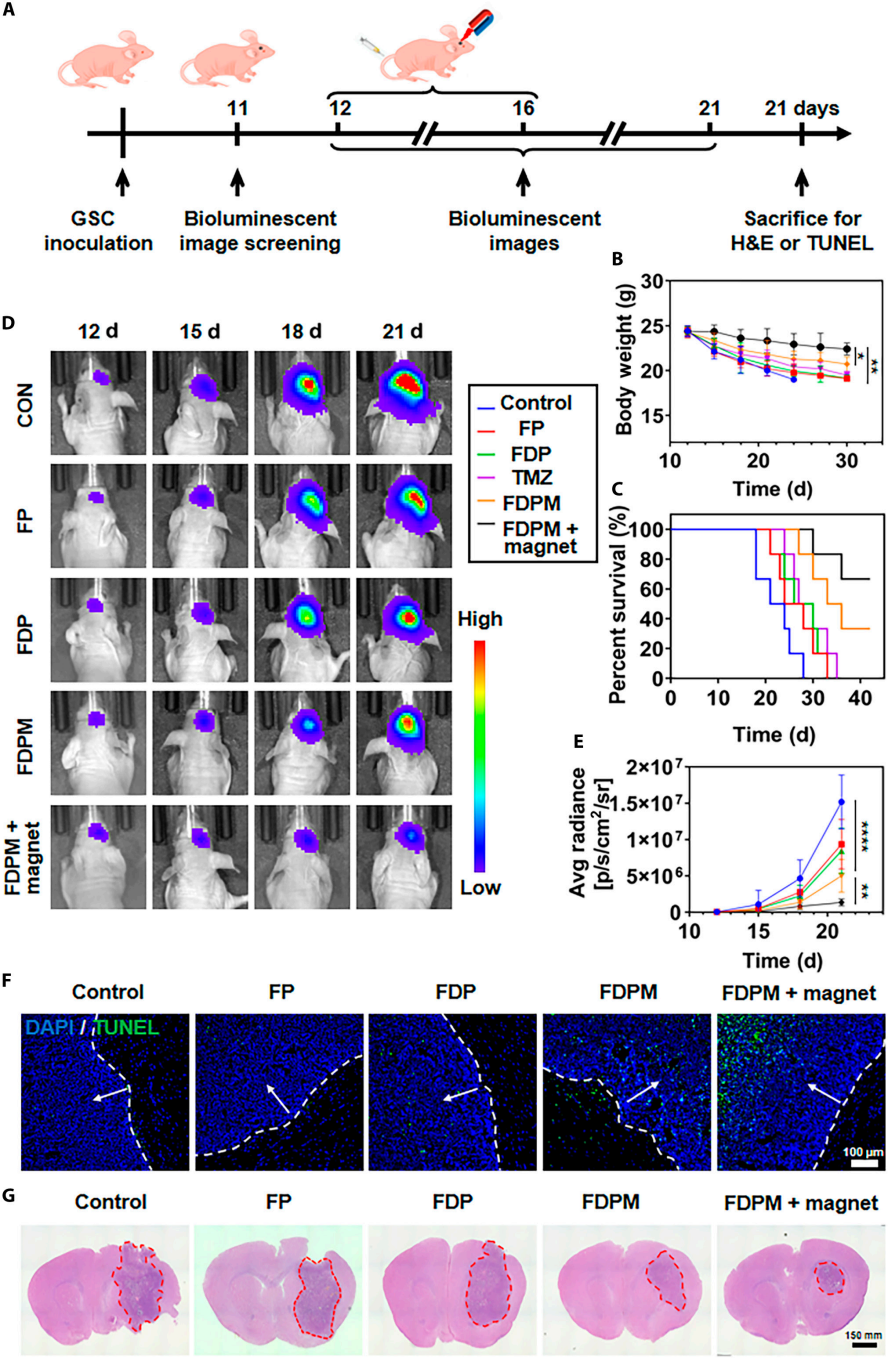
In vivo anti-glioma effects. (A) Schematic illustration of the experimental design. (B) Weight change of tumor-bearing nude mice with different treatments (standardized with 20 mg kg^−1^ FDP). Data are expressed as mean ± SD (*n* = 6). *P < 0.05, **P < 0.01. (C) Survival curves of the tumor-bearing nude mice with different treatments (standardized with 20 mg kg^−1^ FDP) (*n* = 6). Representative bioluminescence assay images (D) and quantified signal intensity (E) of different treatment groups (standardized with 20 mg kg^−1^ FDP). Data are expressed as mean ± SD (*n* = 6). **P < 0.01, ****P < 0.0001. (F) TUNEL staining of residual tumor sites. The white arrows point to the tumor areas. Scale bar, 100 μm. (G) H&E staining analysis of brain tissues in tumor cross-section. The areas circled by red dashed lines indicate residual tumors. Scale bar, 150 mm.

After successfully constructing an in situ glioma model in nude mice using 1 × 10^4^ GSCs per mouse. We applied FDPM combined with magnetic field guidance (30 min of guidance after each administration) to inhibit tumor growth, as shown in the basic procedure in Fig. 7A. Figure 7B and C shows further analysis of the changes in body weight (*P* < 0.01) and the median survival time (*P* < 0.0001) of tumor-bearing nude mice after administration. FDPM with magnetic field guidance significantly reduced the weight loss and extended the median survival period of the glioma-bearing mice. We then transfected GSCs with a lentivirus expressing luciferase, successfully cultivating stable luciferase-expressing ^luc-^GSCs (Fig. S17). With a density of 1 × 10^4 luc-^GSCs per mouse, we built an in situ glioma model in nude mice and administered corresponding treatments to different groups. As shown in Fig. 7D and E, even without the assistance of an immune system, FDPM combined with magnetic field guidance effectively inhibited tumor growth (*P* < 0.0001).

Mice were euthanized on day 22 after modeling, and brain tissues were sectioned for analysis. Figure 7F shows that FDP could only induce tumor apoptosis, while FDPM could induce some tumor cell death, though not efficiently. FDPM markedly induced cell apoptosis at the tumor site under magnetic field guidance. The H&E staining results of the brain sections, shown in Fig. 7G, indicated that FDPM under magnetic field guidance markedly inhibited tumor growth.

In summary, after intravenous administration, FDPM could target and accumulate at the tumor site. With the guidance of a magnetic field, its ability to target the tumor site was greatly enhanced. Additionally, due to the presence of CM, FDPM’s retention in the circulation was also strengthened. This combined magnetic targeting and homologous targeting therapeutic measure could significantly inhibit the malignant growth of tumors caused by GSCs.

### Biosafety evaluation of FDPM

The safety of FDPM as a therapeutic platform is of paramount importance, especially considering its prolonged circulation in the body following intravenous administration. An analysis of the interaction between FDPM and RBCs was crucial to ascertain if it caused any damage to RBCs, such as hemolysis. The results, as presented in Fig. S18, indicated that the nanosystem did not harm RBCs upon contact, thus not leading to hemolysis.

**Fig. 8.**
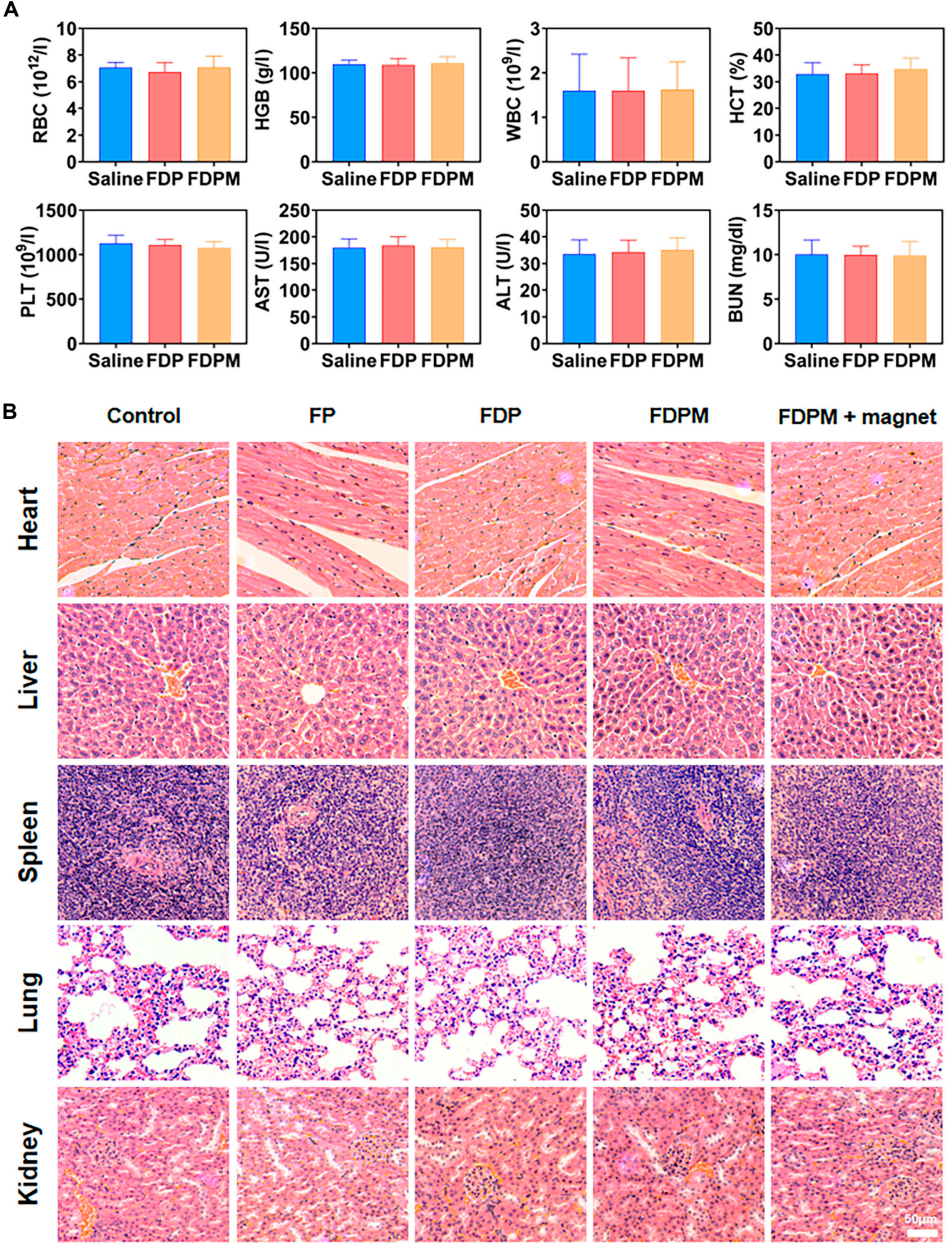
Biosafety evaluation. (A) Blood routine examination of mice after intravenous injection with PBS, FDP, and FDPM. (B) H&E staining analysis of the main organs of mice. Scale bar, 50 μm.

Further analysis of the blood parameters of the treated nude mice showed that all components of the blood were within normal ranges. Additionally, liver and kidney function tests were also completely normal as shown in Fig. 8A. Subsequent analyses involved examining the histopathological sections of various organs from treated nude mice, stained using H&E. These sections revealed normal cellular and structural morphology across different organs, with no evidence of cellular necrosis or apoptosis. This finding, as shown in Fig. 8B, demonstrated that FDPM did not cause apparent organ damage. These results suggested that FDPM is a safe and reliable biomimetic nanosystem.

These comprehensive safety evaluations indicated that FDPM does not induce hemolysis, organ damage, or disruption in blood chemistry and organ function, affirming its suitability for use in therapeutic applications.

## Conclusion

The treatment of gliomas currently necessitates standard surgical resection. However, due to the presence of GSCs, even after the maximal possible surgical resection of tumor cells, the few remaining GSCs can rapidly reactivate tumor growth [40]. Therefore, eradicating GSCs is crucial for the glioma treatment [41].

The biomimetic magnetic responsive nanosystem, FDPM, exhibits significant magnetic targeting and homologous targeting capabilities, enabling it to identify and target GSCs. Initially, after intravenous administration, FDPM can accumulate under the magnetic field attraction located at the tumor site. Next, because of the CM modification, it can remain in living organisms for an extended period and successfully traverse across the BBB. Finally, guided by homologous targeting, it accumulates not only at GSCs but also at tumor cells homologous to GSCs, thereby relying on the DOX to inhibit tumor nucleic acid, killing tumor cells and inhibiting glioma growth.

This strategy offers a new opportunity for antitumor treatment in patients. We believe that it holds the potential to be translated into valuable biomedical applications in the future. The dual-targeting system of FDPM, combining magnetic and homologous targeting, provides a promising approach to address the challenge of killing GSCs, effectively inhibiting tumor growth and improving patient prognosis.

## Data Availability

Data will be made available on request.
