# Supplementary material for: A Magnetic-Responsive Biomimetic Nanosystem Coated with Glioma Stem Cell Membranes Effectively Targets and Eliminates Malignant Gliomas
Source: Biomater Res. 2024 Dec 27;28:0123. doi: 10.34133/bmr.0123 (PMC11676004; doi:10.34133/bmr.0123)
Supplement: Supplementary 1 — Figs. S1 to S18 [file bmr.0123.f1.zip › Supplementary Materials (Clean Version).docx]

**Supplementary Materials**

**A magnetic-responsive biomimetic nanosystem coated with glioma stem cell membranes effectively targets and eliminates malignant gliomas**

Song Deng^1†^, Dekang Nie^1,4†^, Yue Huang^1†^, Yu Yang^5†^, Qianqian Liu^1^, Zesheng Sun^1^, Qiaoji Jiang^4^, Yuejuan Ling^6^, Ya Wen^7^, Jiahua Qu^1^, Jialiang Lin^1^, Yi Wang^3*^, Rongqin Huang^2*^, Jinlong Shi^1*^

^1^ Department of Neurosurgery, Affiliated Hospital of Nantong University, Medical School of Nantong University, No. 20 West Temple Road, Nantong, Jiangsu 226001, P.R China.

^2^ School of Pharmacy, Key Laboratory of Smart Drug Delivery (Ministry of Education), Fudan University, Shanghai, 201203, P.R China.

^3^ Center for Advanced Low-dimension Materials, State Key Laboratory for Modification of Chemical Fibers and Polymer Materials, College of Chemistry, Chemical Engineering and Biotechnology, Donghua University, Shanghai, 201600, P.R China.

^4^ Department of Neurosurgery, The Yancheng Clinical College of Xuzhou Medical University，The First people’s Hospital of Yancheng, Yancheng, Jiangsu 224001, P.R China.

^5^ Department of Neurology, Affiliated Hospital of Nantong University, Medical School of Nantong University, No. 20 West Temple Road, Nantong, Jiangsu 226001, P.R China.

^6^ Institute of Pain Medicine and Special Environmental Medicine, Nantong University, Jiangsu 226019, P.R China.

^7^ Research Center of Clinical Medicine, Affiliated Hospital of Nantong University, No. 20 West Temple Road, Nantong, Jiangsu 226001, P.R China.

^*^ **Corresponding author**

***Jinlong Shi -*** Email: shij@ntu.edu.cn. Department of Neurosurgery, Affiliated Hospital of Nantong University, Medical School of Nantong University, No. 20 West Temple Road, Nantong, Jiangsu 226001, P.R China.

***Rongqin Huang -*** Email: rqhuang@fudan.edu.cn. School of Pharmacy, Key Laboratory of Smart Drug Delivery (Ministry of Education), Fudan University, Shanghai, 201203, P.R China.

***Yi Wang -*** Email: ywang@dhu.edu.cn. Center for Advanced Low-dimension Materials, State Key Laboratory for Modification of Chemical Fibers and Polymer Materials, College of Chemistry, Chemical Engineering and Biotechnology, Donghua University, Shanghai, 201600, P.R China.

† These authors contributed equally to this work.

**Figure S1.** EDX spectroscopy analysis of FDP.

**Figure S2.** Photos of magnetic field generator.

**Figure S3.** The magnetic field response capability of FDPM.

**Figure S4.** (a) XPS patterns of FDP and FP. Enlarged XPS patterns corresponding to N1s (b) and Fe2p3(c).

**Figure S5.** FTIR spectra of FDP and FP.

**Figure S6.** Standard curve of DOX.

**Figure S7.** Photos of FDP dispersion in deionized water, PBS, and simulated body fluid (SBF).

**Figure S8.** Cumulative release curve of FDPM in SBF.

**Figure S9.** Size distribution of FDP and FDPM.

**Figure S10.** Photos of GSC spheres. Scale bar: 50 μm.

**Figure S11.** Fluorescence detection of CD133 and Sox2 in GSCs. Scale bar: 25 μm.

**Figure S12.** Fluorescence detection of CD133 and Nestin in GL261 cells. Scale bar: 100 μm.

**Figure S13.** SDS-PAGE protein analysis of GL261 cells and GSCs.

**Figure S14.** The uptake effects of GSCs on FDPM or FDPM^GL261^. Data are expressed as mean ± SD (n = 3). **P < 0.01.

**Figure S15.** Fluorescence detection of FDPM uptake differences in HUVEC^GFP^ co cultured with GSCs. Scale bar: 50 μm.

**Figure S16.** Survival curves of the tumor-bearing nude mice with different modeling methods.

**Figure S17.** Bioluminescence assay images of luciferase-expressing ^luc-^GSCs.

**Figure S18.** The percentage of hemolysis with different concentrations of FDPM. Data are expressed as mean ± SD (n = 3).
